# 

**Published:** 1892-07

**Authors:** 


ESTABLISHED 1855. SUBSCRIPTION, $2.00.

### ATLANTA MEDICAL AND SURGICAL JOURNAL.

OLD SERIES, VOL. XXVI. }

NEW SERIES, VOL. IX. } ATLANTA, GA., JULY, 1892. No. 5.

LUTHER B. GRANDY, M. D., MANAGING EDITOR.

M. B. HUTCHINS, M. D. BUSINESS MANAGER.

